# Recognition-induced destabilization: controlled release from molecularly imprinted chitosan nanoparticles *via* specific, non-catalytic enzyme recognition

**DOI:** 10.1039/d5ra05081b

**Published:** 2025-10-31

**Authors:** Mutasem O. Taha, Isra Dmour, Ramzi Mukred Saeed, Taqwa Alfararjeh, Inshad Jum'h, Lina A. Dahabiyeh

**Affiliations:** a Department of Pharmaceutical Sciences, School of Pharmacy, The University of Jordan Amman 11942 Jordan mutasem@ju.edu.jo; b Department of Pharmaceutics and Pharmaceutical Technology, Faculty of Pharmaceutical Sciences, The Hashemite University Zarqa 13133 Jordan; c Helmholtz Institute for Translational Oncology, HI-TRON Mainz Mainz 55131 Germany; d Division of Personalized Immunotherapy, DKFZ Heidelberg Germany; e School of Basic Sciences and Humanities, German-Jordanian University (GJU) Amman 11180 Jordan

## Abstract

This work introduces a novel paradigm for stimuli-responsive drug delivery: recognition-induced destabilization, where specific molecular recognition—without enzymatic catalysis—triggers nanoparticle disassembly. We engineered chitosan-phthalate nanoparticles (NPs) *via* molecular imprinting using lysozyme or α-glucosidase as templates. Critically, these enzymes do not catalytically degrade deacetylated, cross-linked, chitosan NPs enabling isolation of the recognition effect. Upon recognition by their respective enzyme, the imprinted nanoparticles (nanoMIPs) exhibited selective structural destabilization confirmed by Dynamic Light Scattering (DLS), while non-imprinted controls remained stable. This recognition event facilitated highly specific, on-demand release of encapsulated ciprofloxacin, achieving >90% release compared to <11% from controls. These findings demonstrate that imprint-guided recognition, coupled with proximity-induced microstructural degradation, can induce catastrophic mechanical failure of nanoMIPs and trigger drug release. The high specificity, stability, and responsiveness of this platform highlight its potential for translation into targeted therapies, biosensing, and diagnostic applications. Future studies will explore *in vivo* performance in enzyme-rich microenvironments such as infection and inflammation sites.

## Abstract 2

A key to destruction: specific, non-catalytic protein recognition is harnessed to trigger the mechanical failure of molecularly imprinted nanoparticles, enabling highly specific, on-demand release of an encapsulated payload.

## Graphical abstract

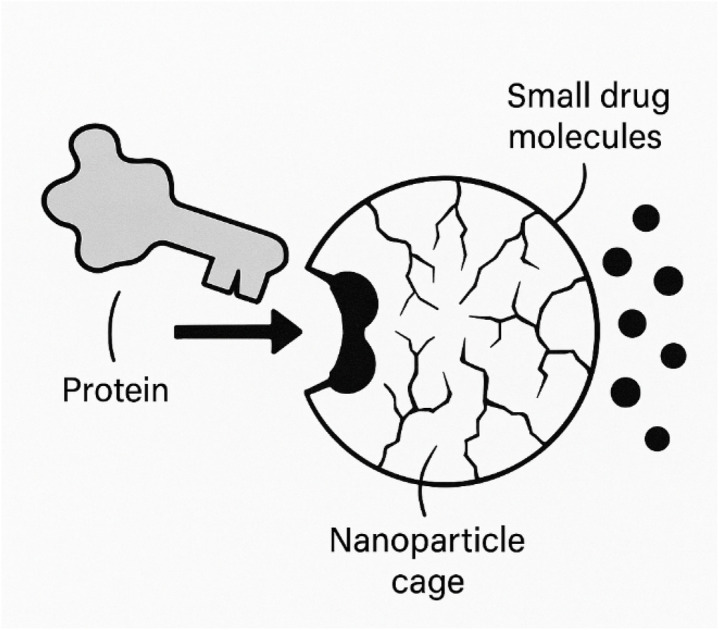

## 1. Introduction

The development of “smart” drug delivery systems that can respond to specific biological cues remains a central challenge in biomedical research.^1^ Stimuli-responsive nanoparticles are particularly attractive because they can release therapeutic agents selectively at diseased sites, thereby improving efficacy while minimizing systemic toxicity. Conventional stimuli include pH, temperature, redox potential, and enzymatic activity, each offering varying levels of specificity and control effects.^2–6^ Among these, enzyme-responsive systems have drawn considerable attention because enzymes are tightly associated with disease states and pathological microenvironments.^7^

However, most existing enzyme-responsive materials rely on the enzyme's catalytic function to degrade polymer backbones, leading to nanoparticle disassembly.^8^ This strategy, while effective, often suffers from limited specificity, since many enzymes share overlapping catalytic substrates, and degradation is typically slow and uncontrolled.^9^ A more sophisticated approach would be to design systems that respond not to enzymatic cleavage but to the mere presence of a specific enzyme, transforming molecular recognition into a physical destabilization trigger.

Molecular imprinting technology provides a promising platform for this purpose. By polymerizing functional monomers around a target molecule, molecularly imprinted polymers (MIPs) form cavities that are chemically and sterically complementary to the template.^10–12^ MIPs have been widely applied in drug delivery, diagnostics, biosensing, and targeted therapies,^13–27^ with recent advances demonstrating protein-imprinted nanoparticles (nanoMIP) capable of selective cell targeting, controlled release, and *in vivo* recognition of disease biomarkers.^22,24,28^ Despite this progress, molecular imprinting has never been exploited to induce mechanical failure of nanoparticles through recognition events alone.

In this work, we introduce a new paradigm—recognition-induced destabilization—where specific, non-catalytic recognition of an enzyme within imprinted cavities is sufficient to trigger nanoparticle disassembly and controlled drug release. The system is built on a chitosan-phthalate (CS-PH) matrix, whose extraordinary stability we previously established.^29,30^ Chitosan was chosen not only for its biocompatibility, biodegradability, versatile chemistry, and mucoadhesive properties,^29–40^ but also because phthalate conjugation followed by dual ionotropic/covalent crosslinking yields nanoparticles highly resistant to nonspecific degradation, premature drug leakage, or environmental stress.^29,30^ Indeed, CS-PH nanoparticles normally remain intact even in enzyme-rich or physiologically challenging conditions, making them an exceptionally stringent platform for testing recognition-driven effects.^30^

Against this backdrop of exceptional stability, we asked whether destabilization could be triggered purely by molecular recognition. To this end, molecularly imprinted CS-PH nanoparticles (MICNPs) were fabricated using either lysozyme or α-glucosidase as templates. These enzymes were deliberately selected for two key reasons. First, neither enzyme is capable of cleaving the β-1,4-glycosidic bonds of highly deacetylated, cross-linked chitosan backbone used in our study,^41–44^ ensuring that any nanoparticle destabilization arises exclusively from molecular recognition rather than enzymatic degradation of the polymer backbone. Second, both enzymes are clinically relevant: lysozyme is a ubiquitous innate immune enzyme enriched at infection and inflammation sites,^42^ while α-glucosidase is a metabolic enzyme with therapeutic importance in gastrointestinal and metabolic disorders.^44^ By employing two functionally distinct, non-degradative enzymes, we aimed to demonstrate that recognition-induced destabilization is a generalizable mechanism rather than an effect restricted to a single enzyme system.

We hypothesized that enzyme binding within the imprinted cavities would impose proximity-driven microstructural stress on the otherwise inert CS-PH network, leading to localized disruption and nanoparticle destabilization.

**Fig. 1:**
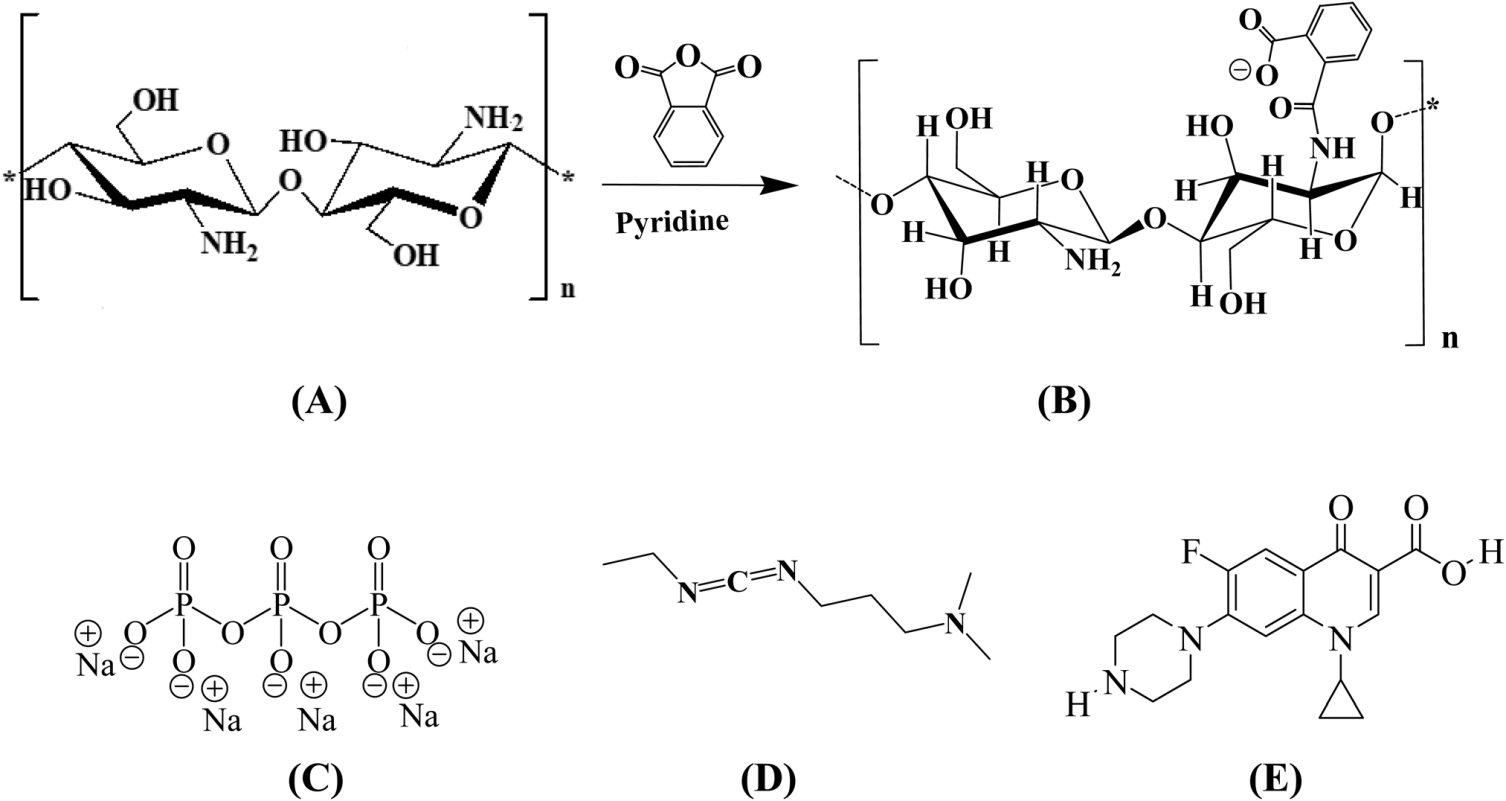
Chemical structure of (A) chitosan (CS), (B) chitosan phthalate (CS-PH), the combination of (A) and (B) represents the synthetic preparation of CS-PH, (C) tripolyphosphate sodium (TPP), (D) *N*-ethyl-*N*′-(3-dimethylaminopropyl) carbodiimide hydrochlorides (EDC), (E) ciprofloxacin.

**Fig. 2:**
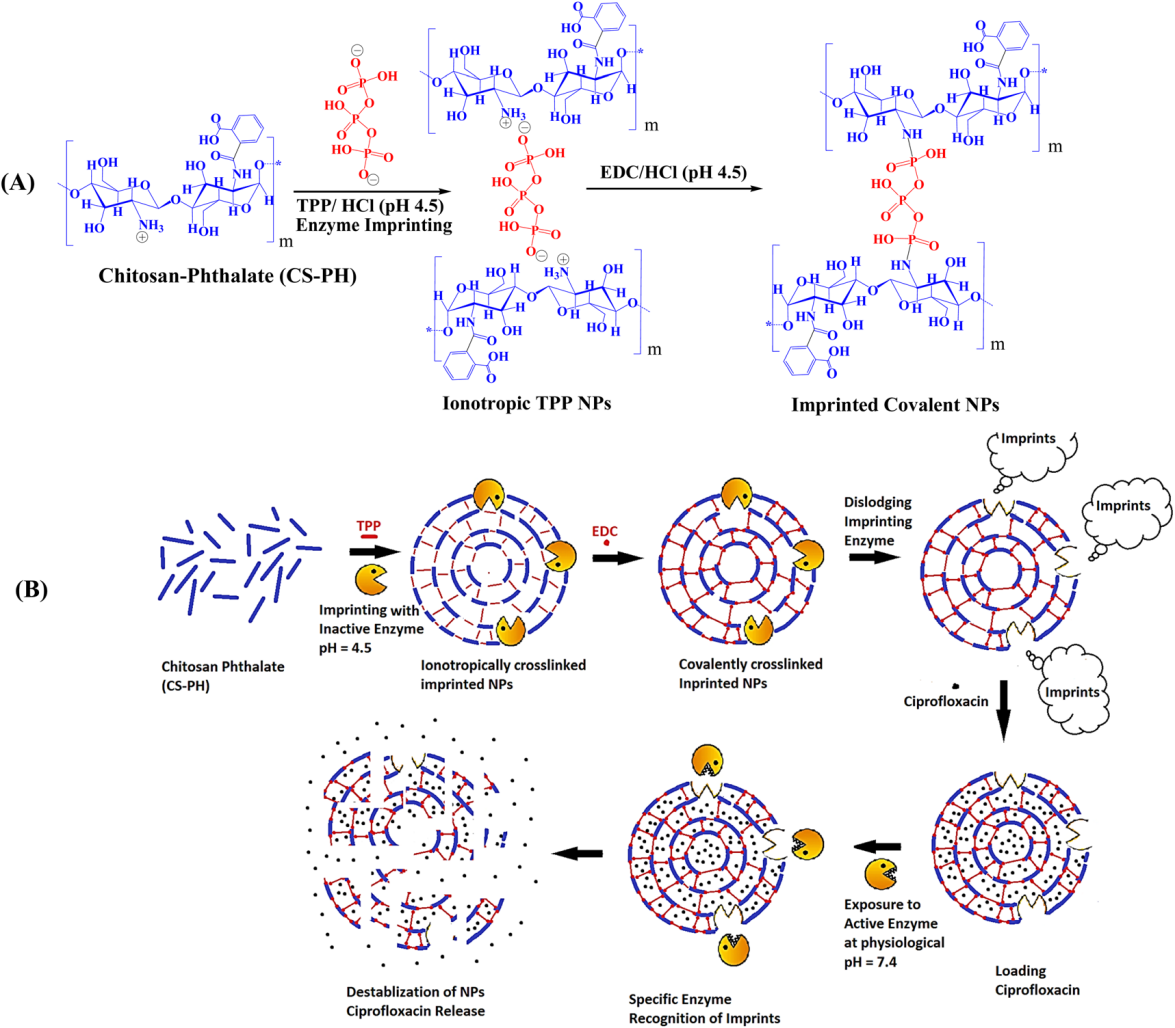
(A) Ionotropic/covalent crosslinking of CS-PH with TPP and EDC. (B) Schematic representation of nanoparticles fabrication process including imprinting with digestive enzyme(s), nanoparticles loading, nanoparticles destabilization and drug release upon exposure to the active form of the imprinting enzyme.

To test this hypothesis, MICNPs were prepared *via* our robust dual crosslinking method^29,30^ (Fig. 1 and 2), characterized extensively, and then challenged in ciprofloxacin release assays. As expected, non-imprinted controls remained stable and released less than 11% of their cargo. In striking contrast, imprinted nanoparticles, despite their inherent robustness, underwent selective, enzyme-triggered swelling and destabilization, releasing over 90% of the encapsulated drug in the presence of their cognate enzyme. Thus, molecular recognition alone was able to overcome the extraordinary baseline stability of CS-PH nanoparticles. This finding establishes a fundamentally new mechanism for smart drug delivery and expands the scope of molecular imprinting into recognition-driven mechanical responses.

## 2. Materials and methods

### 2.1. Materials

All chemicals were purchased from respective companies (in brackets) and were used without pretreatment or purification. Low molecular weight chitosan (Sigma-Aldrich, USA), phthalic anhydride (Fluka, Switzerland), chicken egg lysozyme (Sigma-Aldrich, USA), α-glucosidase from *Saccharomyces cerevisiae* (Sigma-Aldrich, USA), sodium triphosphate pentabasic (Sigma-Aldrich, Germany), *N*-ethyl-*N*′-(3-dimethylaminopropyl) carbodiimide hydrochlorides (EDC) (Caymanchem, USA), potassium chloride (Sigma-Aldrich, USA), potassium di-hydrogen orthophosphate/di-sodium hydrogen phosphate to prepare phosphate-buffered saline (PBS, pH 7.4, Sigma-Aldrich, USA), pyridine (Labchem, USA), sodium hydroxide (Rasayan Laboratory, India), hydrochloric acid (37%) (Carlo Erba, Spain), absolute ethanol and acetone (analytical grade, Carlo Erba, Spain). Water used for particle size measurements was ultrapure water (Millipore®, conductivity = 0.05 μs cm^−1^), while deionized water was used for other measures. Amicon® Ultra centrifugal filters (Merck Millipore, USA), ciprofloxacin base was obtained as a kind gift from Dar Al-Dawa (Na'or, Jordan).

### 2.2. Synthesis of chitosan phthalate

Chitosan-phthalate (CS-PH) with a 10% degree of substitution was synthesized as we previously described with minor modifications.^29,30,45^ Low molecular weight chitosan (1.00 g, corresponding to 6.23 mmol of glucosamine) was dissolved in 50 mL of 0.37% (v/v) aqueous HCl under continuous stirring at room temperature. Separately, phthalic anhydride (0.092 g, 0.623 mmol) was dissolved in 5 mL of pyridine and added dropwise to the chitosan solution while maintaining constant stirring. The reaction mixture was stirred for 45 minutes, and the pH was adjusted to 7.0 using 1.0 N NaOH. The CS-PH derivative was precipitated by adding acetone, and the resulting precipitate was collected by filtration. The precipitate was washed three times with 100 mL of absolute ethanol, followed by 100 mL of acetone, and then dried in a hot air oven at 40 °C for 48 hours. The dried product was stored in an airtight container at room temperature.

### 2.3. Fabrication of CS-PH molecularly imprinted and non-imprinted nanoparticles

The preparation of CS-PH nanoparticles (NPs) was carried out using the syringe-dropping method as we previously described.^29,30,46^ A 0.1% w/v CS-PH solution was prepared by dissolving CS-PH in aqueous HCl (4.8 mM) under continuous stirring overnight. The solution was filtered through Whatman® filter paper (15 cm) to remove insoluble polymer. The amounts of TPP and EDC used were determined empirically based on our previous work to achieve optimal nanoparticle formation (*i.e.*, the formation of a stable colloidal dispersion with the desired size and charge characteristics).^29,30^

For the non-imprinted NPs, freshly prepared and filtered aqueous TPP solution (*ca.* 430 μL, 0.4% w/v in Milli-Q® water at room temperature) was added dropwise to the CS-PH solution (5 mL) under vigorous stirring at 25 °C until a hazy dispersion formed. Stirring was continued for 1 minute, followed by the gradual addition of EDC powder (25 mg). The mixture was stirred for an additional minute and then left to react overnight at preparation pH (4.5) with EDC. The next day, the NPs dispersion underwent sonication (Soner 203H Rocker, Taiwan) for 60 seconds to promote surface desorption of excess EDC and by-products. Subsequently, 4 mL of the dispersion was transferred to an Amicon® tube (molecular weight cutoff 100 kDa) and centrifuged at 4500 rpm at 20 °C for 30 minutes. The concentrated dispersion was gently mixed with a pipette and re-centrifuged under the same conditions for an additional 30 minutes. This process was followed by washing the NPs twice with 1 mL PBS (pH 4.5), each time involving gentle mixing and centrifugation under the same conditions. After the final centrifugation step, approximately 150 μL of NPs suspension was obtained. These washing/centrifugation cycles were intended to remove excess EDC and by-products. The concentrated NPs dispersion was transferred to a glass vial and left overnight at the same pH for subsequent manipulations. The resultant NPs were used for characterization using DLS, IR, DSC and as blanks for drug release studies.

For the imprinted nanoparticles, a similar procedure was followed with the inclusion of the specific enzyme (lysozyme or α-glucosidase) during crosslinking with TPP. The imprinting enzyme solution (1.5% w/v) was freshly prepared by dissolving 22.5 mg of the enzyme in 1.5 mL HCl (4.8 mM in Milli-Q® water at room temperature). The TPP solution was added dropwise in four steps: in the first three steps, TPP alone was added (130 μL in the first addition, followed by 100 μL in each of the subsequent two additions) to the CS-PH solution under vigorous stirring, with each addition taking approximately 4 seconds. In the fourth step, 100 μL of the enzyme solution and 100 μL of the TPP solution were simultaneously added from separate micropipettes. Stirring was continued vigorously for 1 minute, and EDC was subsequently added as described for non-imprinted NPs. The imprinted NPs were left standing to react overnight at room temperature and preparation pH of 4.5. This acidic pH is a critical design element, as it ensures the imprinting enzyme remains in an inactive state. This prevents any enzymatic degradation of the chitosan matrix during fabrication and allows stable imprints to form.

The following day, the NPs dispersion underwent sonication (Soner 203H Rocker, Taiwan) for 60 seconds to facilitate surface desorption of excess enzyme, EDC and EDC by product. The dispersion (4 mL) was subjected to centrifugation and washing cycles in Amicon® tubes (molecular weight cutoff 100 kDa), as previously described for non-imprinted NPs. These cycles were designed to remove excess EDC, free enzyme, EDC by-products, and dislodging imprinting enzyme molecules from the surface imprints. This repetitive washing is essential to completely dislodge the imprinting enzyme molecules, as incomplete removal can cause the resulting imprinted NPs to destabilize and release their contents prematurely, even without the presence of the corresponding active enzyme in the dissolution medium. Finally, the concentrated NPs dispersion (150 μL) was transferred to a glass vial and left overnight at the same pH (*i.e.*, 4.5) for subsequent manipulations.

### 2.4. Drug loading and release

A saturated ciprofloxacin loading solution was prepared by adding excess ciprofloxacin base (3.7 g) to a fixed volume of phosphate-buffered saline (PBS, 100 mL, pH 7.4) with continuous stirring for 4 hours at room temperature. The resulting suspension was then filtered through Whatman® filter paper (15 cm diameter) to obtain a clear, saturated solution. Aliquots of this solution (150 μL each) were added in equal volumes to the nanoparticle (NP) dispersions (either imprinted or non-imprinted, maintained in PBS at pH 4.5). The mixtures were allowed to stand undisturbed overnight at 25 °C (strictly controlled). Following incubation, the total dispersion volume was adjusted to 4 mL using PBS (pH 7.4) for subsequent drug release studies. The release experiments were conducted at pH 7.4 as this represents a standard physiological pH at which the template enzymes are in their catalytically active conformation. All procedures involving ciprofloxacin were conducted under protection from direct light to prevent drug degradation. Drug-free NP dispersions (blanks) were prepared using the same protocol, omitting the addition of ciprofloxacin.

The samples were aliquoted into 2 mL portions and transferred to quartz cuvettes. These cuvettes were incubated in a shaking water bath (Thermo Scientific, USA) at 100 rpm and 37 °C. Ciprofloxacin absorbance was continuously monitored at its maximum absorbance wavelength (*λ*_max_ = 273 nm) using a UV-visible spectrophotometer (Cary 1E, Varian, USA) until a steady-state baseline was achieved (indicative of excess unloaded ciprofloxacin).

Subsequently, 30 μL of one of the following solutions—lysozyme, α-glucosidase (20 μg dissolved in 1.5 mL PBS, pH 7.4), or PBS (pH 7.4) alone—was added to the 2 mL nanoparticle (NP) sample cuvettes. The mixture was gently homogenized using a micropipette, and the cuvettes were returned to the shaking water bath at 37 °C. Absorbance measurements at *λ*_max_ = 273 nm were recorded at predetermined time intervals. For plotting release profiles, the released ciprofloxacin concentrations were subtracted from the corresponding baseline ciprofloxacin concentrations.

The procedure was applied to both molecularly imprinted and non-imprinted nanoparticles. Drug-free NPs, prepared using the same protocol, were employed to zero the spectrophotometer. All drug release experiments were conducted in triplicate to ensure data reproducibility and reliability. The concentrations were determined based on appropriately drawn concentration–absorbance calibration curves at corresponding pH and temperature conditions.

### 2.5. Encapsulation capacities of nanoparticles

Baseline ciprofloxacin concentrations in the release medium, measured before enzyme addition (representing excess unloaded ciprofloxacin), were converted to amounts (μg) and subtracted from the ciprofloxacin amounts in the corresponding loading solution (measured after filtration of the saturated ciprofloxacin solution used for loading, as described previously). This difference was used to calculate the encapsulation capacity of NPs using the formula:
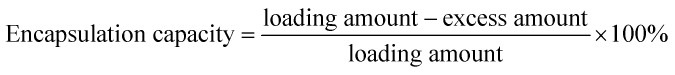


Ciprofloxacin concentrations were determined using concentration–absorbance calibration curves established at the relevant pH and temperature conditions. All measurements were performed in triplicate to ensure reproducibility and reliability.

### 2.6. Characterization of NPs

#### 2.6.1. Dynamic light scattering (DLS)

Zetasizer Nano ZS (Zetasizer Nano ZS, Malvern Instruments, UK) was used to measure the particle size, PDI, and zeta potential of the prepared NPs. Additionally, DLS was used to monitor NPs size distribution before and after drug loading and release studies. The following parameters were assumed in the calculations: media viscosity = 0.8872 cP, dielectric constant = 78.5, and the scattering angle used for the analysis was 90° at 25 °C. Measurements were run in triplicate, and average mean diameter ± SD was calculated. Zetasizer software (version 7.11) was used to process all measurements.

#### 2.6.2. Differential scanning calorimetry (DSC) studies

Thermal analysis of the lyophilized imprinted and non-imprinted NPs at prepared at pH 4.5 and 7.4 (PBS) was conducted *via* Differential Scanning Calorimetry (DSC) utilizing a DSC 823e Mettler Toledo (Switzerland). Samples (weighing 5–7 mg) were placed in aluminum sample pans and hermetically sealed. Each sample was heated from 25 to 350 °C at a rate of 10 °C min^−1^ under a nitrogen purge, utilizing an empty sealed pan to establish a baseline. Prior to samples scanning, calibration for temperature and heat flow was performed using indium.

#### 2.6.3. Infrared (IR) spectroscopy

IR spectra were collected for lyophilized imprinted and non-imprinted NPs using a Thermo DS® Attenuated Total Reflection (ATR) Spectrometer (Germany) at ambient temperature over a wavelength range of 4000 to 400 cm^−1^ with a resolution of 1.0 cm^−1^. ATR measurement was performed without any prior treatment.

#### 2.6.4. Transmission electron microscopy (TEM)

The morphology of non-imprinted (NINPs) and lysozyme-imprinted nanoparticles (Lys-MIPs) was examined by transmission electron microscopy (TEM) using a Morgagni™ FEI 268 instrument (Holland) equipped with a MegaView camera. To minimize aggregation artifacts during imaging, freshly prepared nanoparticle suspensions were vortexed prior to grid deposition. A drop of each suspension was placed onto carbon-coated copper grids, allowed to settle, and dried at room temperature for 1 hour before imaging.

#### 2.6.5. Atomic force microscopy (AFM) studies

The topography of the imprinted and non-imprinted NPs surfaces was investigated using AFM techniques. For the purpose of AFM imaging, highly oriented pyrolytic graphite (HOPG) substrates were used. The samples were prepared by drop casting of the NPs on the substrate. The samples were then left in air to allow for complete evaporation of the drop. After that, the samples were loaded into the microscope. All AFM images were recorded in FM-AFM with constant oscillation amplitude of 1 nm and a constant scan speed of 0.25 Hz. The images were recorded at frequency shifts ranging from 2 to 6 Hz with the PLL bandwidth limited to 150 Hz. Resolution for topography measurements was 256 × 256 points.

### 2.7. Statistical analysis

All experimental data were expressed as mean and standard deviation (SD) from triplicate experiments (*n* = 3). Microsoft Excel Software 2007 (Microsoft Corp., Redmond, WA, USA) was utilized to compute means and standard deviations for size, zeta potential, PDI, encapsulation efficiency, and cumulative release amounts, as well as to generate graphs.

## 3. Results and discussion

### 3.1. Synthesis and physicochemical validation of imprinted nanoparticles

The synthesis of chitosan-phthalate (CS-PH) and its subsequent formulation into nanoparticles *via* ionotropic crosslinking with TPP followed by covalent crosslinking with EDC was successfully achieved as we described earlier.^29,30^ Physicochemical characterization revealed key differences between non-imprinted (NINPs) and molecularly imprinted nanoparticles (MIPs). The average particle sizes, ranging from approximately 176 nm for lysozyme imprinted NPs (Lys-MIPs, after template removal) to 233 nm for NINPs (Table 1), are generally within a range suitable for various drug delivery applications, including oral administration or potentially exploiting the enhanced permeability and retention effect in tumor tissues if such targeting were intended.^29,30^

**Table 1:** Size and charge characteristics of lysozyme-imprinted NPs at preparation pH

Metrics^a^	NINPs at preparation pH	Lys-MIPs	
		Before dislodging imprinting lysozyme	After dislodging imprinting lysozyme
Average size (range, nm)	233.5 (218.6–272.3)	154.1 (135.3–183.5)	175.6 (152.2–196.5)
Size standard deviation (nm)	19.97	17.97	17.44
Average PDI (range)	0.60	0.44	0.04
PDI standard deviation	0.09	0.21	0.05
Average zeta (range, mV)	15.6 (14.1–16.9)	15.9 (15.0–16.5)	15.3 (14.0–16.6)
Zeta standard deviation	1.12	0.62	1.52

a The values represent the average of at least triplicate measurements.

A particularly significant finding is the remarkably low polydispersity index (PDI) of 0.04 observed for Lys-MIPs after the removal of the imprinting enzyme, compared to a PDI of 0.60 for NINPs. This low PDI value indicates the formation of a highly monodisperse population of nanoparticles. The process of imprinting, which involves TPP/EDC crosslinking of CS-PH polymeric chains around template molecules, followed by template removal and washing steps, appears to refine the nanoparticle population, leading to enhanced uniformity. Such monodispersity is highly desirable for drug delivery systems as it can lead to more predictable *in vivo* pharmacokinetics, consistent biodistribution, and reproducible drug release profiles, thereby enhancing the overall reliability and efficacy of the therapeutic system.

The initial smaller size of Lys-MIPs before template removal (154.1 nm) compared to NINPs (233.5 nm) suggests that the presence of the enzyme template during nanoparticle formation influences the condensation and arrangement of the polymer chains. The slight increase in size after template removal (to 175.6 nm) might be due to the creation of vacant cavities and minor matrix relaxation. When the nanoparticles were evaluated at pH 7.4 (the conditions for release studies, Table 2), their distinct size characteristics were maintained. Lys-MIPs remained relatively small and uniform, with an average size of 159.1 nm and a PDI of 0.25. In contrast, NINPs and α-glucosidase-imprinted nanoparticles (Glu-MIPs) were larger, with average sizes of 259.7 nm and 265.0 nm, respectively. The positive zeta potential values (around +15 mV) observed for all nanoparticle formulations are consistent with the presence of protonated amino groups on the chitosan backbone at the acidic preparation pH of 4.5.

**Table 2:** Encapsulation capacities and size properties of different NPs at pH 7.4

Nanoparticles	Encapsulation capacity^a^	Size properties at pH 7.4^a^^,^^b^	
		Size (nm)	PDI
NINPs	26.3% (±2.7)	259.7 (±35.6)	0.39 (±0.02)
Lys-MIPs	52.2% (±4.0)	159.1 (±31.1)	0.25 (±0.04)
Glu-MIPs	55.8% (±7.6)	265.0 (±8.1)	0.48 (±0.08)

a The values represent the average of at least triplicate measurements.

b Upon exposure over approximately 45 minutes.

Perhaps one of the most compelling advantages conferred by the imprinting process is the substantial improvement in drug encapsulation capacity. Both Lys-MIPs (52.2%) and Glu-MIPs (55.8%) demonstrated an approximately twofold higher ciprofloxacin encapsulation capacity compared to NINPs (26.3%) (Table 2).

The creation of specific template-shaped cavities during imprinting is primarily intended for molecular recognition. However, these cavities, or the overall altered polymer network architecture formed in the presence of the bulky enzyme template, may also provide additional or more favorable spaces for the entrapment of drug molecules like ciprofloxacin. It is plausible that the imprinting process influences the porosity and internal structure of the nanoparticles in a way that extends beyond the specific binding sites, creating a matrix more conducive to drug incorporation. This dual benefit of enhanced specificity (discussed later) and significantly increased drug loading capacity makes the molecular imprinting strategy particularly attractive for developing advanced drug delivery vehicles.

**Fig. 3:**
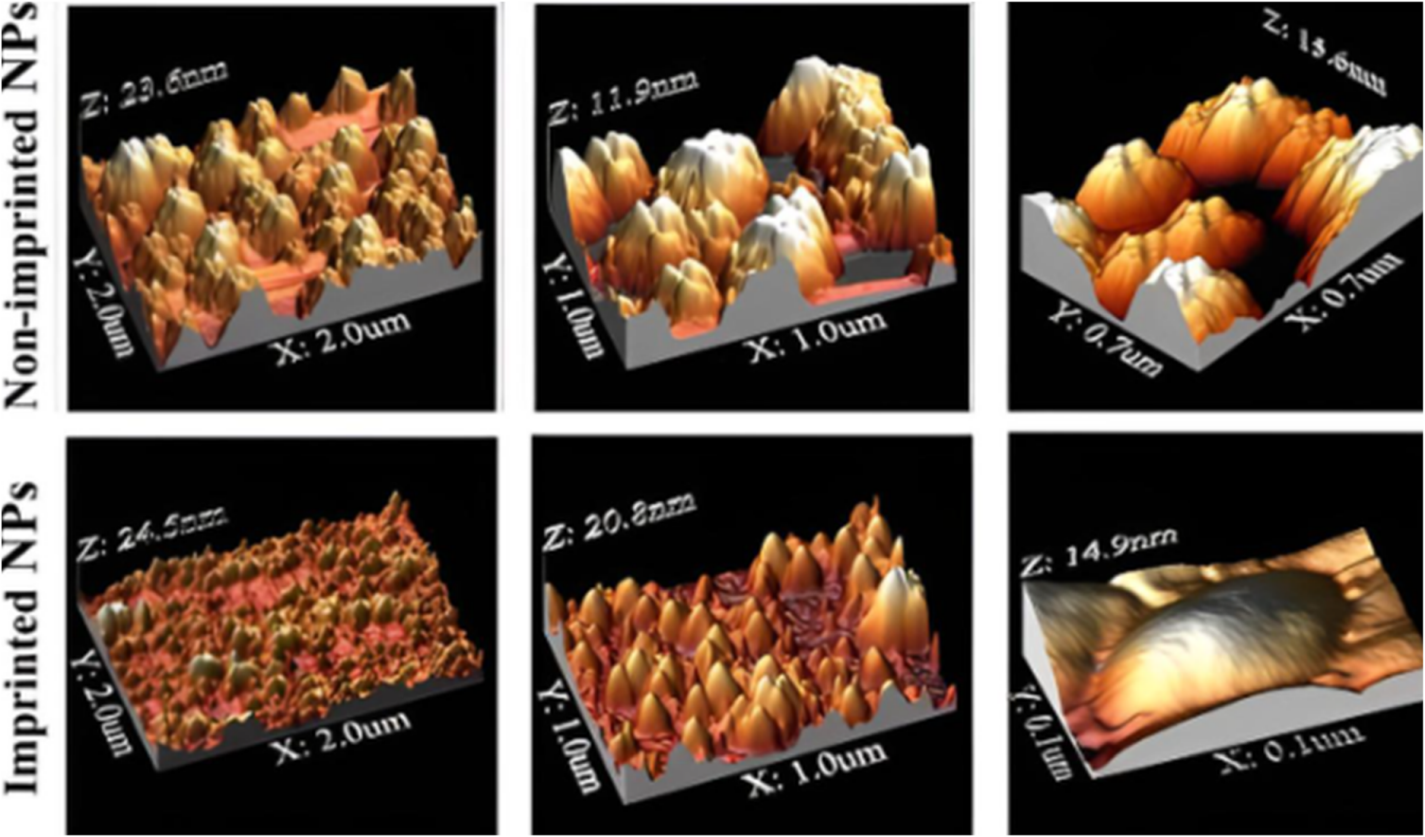
Atomic force microscopy (AFM) images.

The distinct physical properties conferred by imprinting are reflected in the nanoparticle morphology. Atomic Force Microscopy (AFM) imaging revealed clear morphological differences between non-imprinted nanoparticles (NINPs) and lysozyme-imprinted nanoparticles (Lys-MIPs) (Fig. 3). NINPs appeared as aggregated clusters with relatively smooth, uniform surfaces, whereas Lys-MIPs exhibited more granular, textured surfaces with higher roughness. These changes are attributed to the imprinting process, where the presence and subsequent removal of the lysozyme template generate surface cavities and structural complexity. The distinct AFM topography provides direct visual evidence of imprint formation and supports the role of these features in specific enzyme recognition, destabilization, and drug release. These AFM findings align with previous reports showing that molecular imprinting introduces measurable surface changes in nanoparticles.^47^

**Fig. 4:**
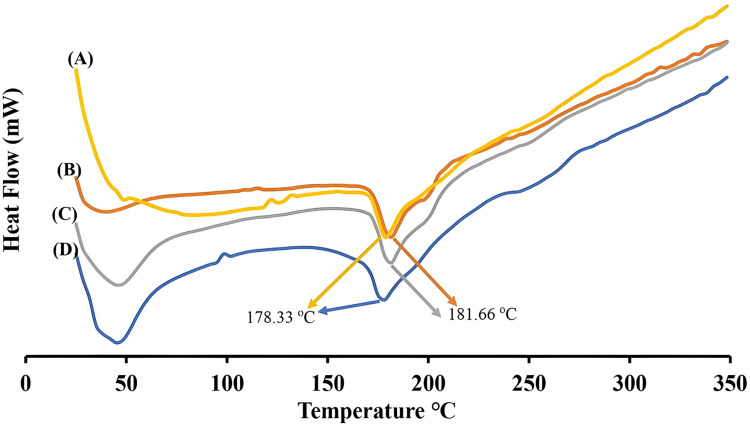
Differential scanning calorimetry (DSC) thermal properties of various nanoparticles (NPs). (A) Lysozyme-imprinted NPs lyophilized immediately following preparation (pH 4.5 and 37 °C). (B) Non-imprinted NPs lyophilized immediately following preparation (pH 4.5 and 37 °C). (C) Non-imprinted NPs lyophilized after 2 hours of incubation with lysozyme in dissolution medium (pH 7.4 and 37 °C). (D) Lysozyme-imprinted NPs lyophilized after 2 hours of incubation with lysozyme in dissolution medium (pH 7.4 and 37 °C).

In addition to surface morphology, the thermal properties of the nanoparticles were also altered by the imprinting process. Differential Scanning Calorimetry (DSC) was used to investigate the thermal properties and stability of the nanoparticles (Fig. 4). The thermograms reveal a broad endothermic peak at approximately 180 °C, attributable to thermal melting of the NPs matrices. For Lys-MIPs, imprinting with lysozyme caused a noticeable shift in the melting peak temperature from 181.66 °C in non-imprinted nanoparticles (traits B and C in Fig. 4) to 178.33 °C in imprinted nanoparticles (traits A and D in Fig. 4). Interestingly, this shift occurred regardless of exposure to lysozyme in the dissolution medium, suggesting that the destabilizing effect of lysozyme on Lys-MIPs is too subtle to be detected by DSC. The slight, but consistent, reduction in the melting temperature implies that the imprinting process marginally compromises the structural integrity of the nanoparticle matrix. This effect may result from the formation of surface vacant cavities that create a slightly weaker hydrogen-bonding networks within the NPs polymeric shell—a phenomenon previously reported for molecularly imprinted polymers, where the presence of template molecules and imprinting conditions alter porosity, surface morphology and thermal transitions (DSC) of the host polymer.^48,49^

Collectively, the physicochemical data establish that the molecular imprinting process successfully yields a uniform population of nanoparticles with distinct morphological and thermal properties that are structurally “primed” for recognition-based responses.

### 3.2. Recognition-induced destabilization: a “lock-and-key” structural response

**Fig. 5:**
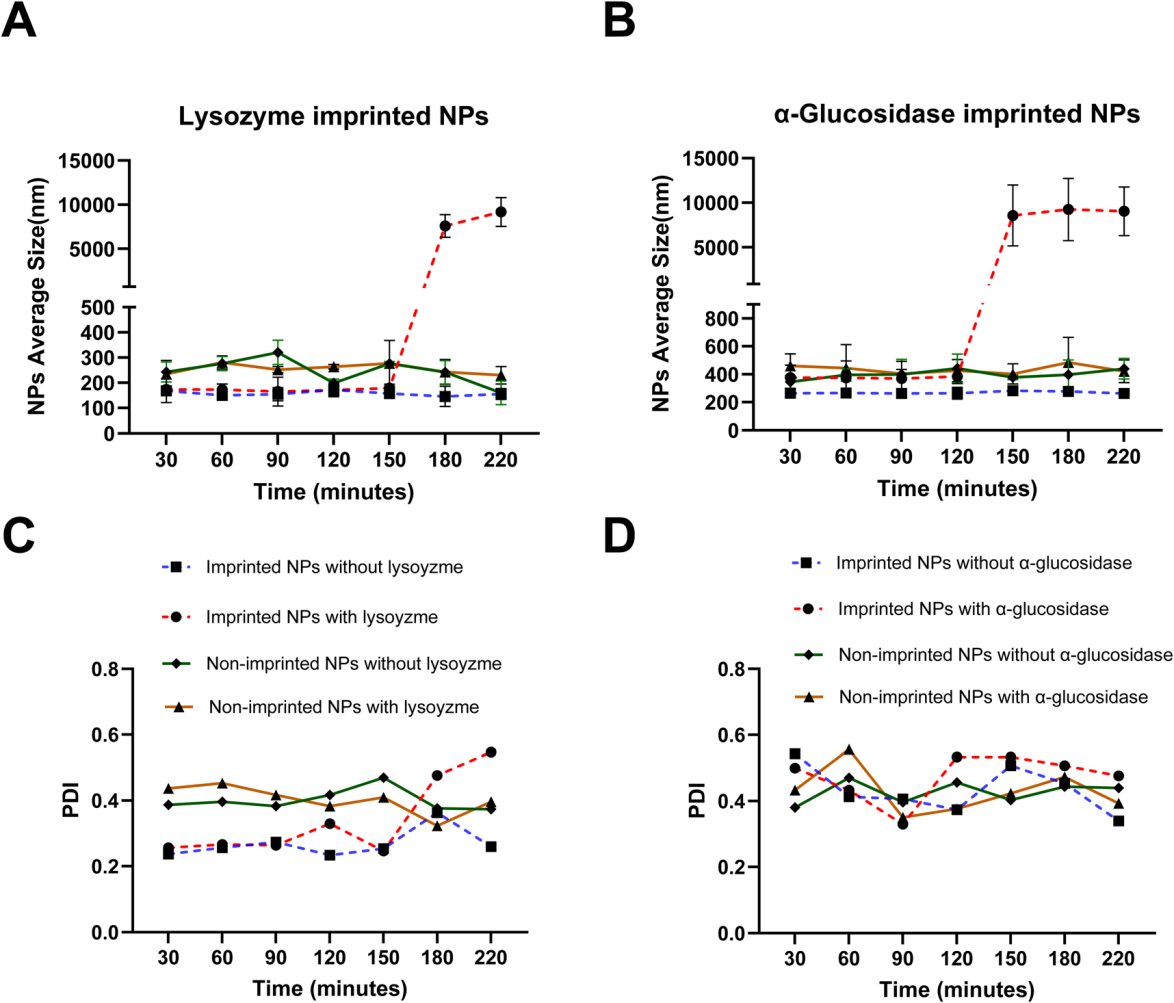
Effect of cognate enzymes (Lysozyme, (A and C), *versus* α-glucosidase, (B and D)) in the dissolution media on NPs sizes (A and B) and polydispersity index (PDI, C and D). Each point represents the average of three separate experiments (*n* = 3). Error bars represent the standard deviation of the measurements. All experiments were performed at 37 °C.

The responsiveness of the imprinted nanoparticles to their respective imprinting enzymes was investigated by monitoring changes in their hydrodynamic size over time using DLS at 37 °C. Fig. 5 shows the size profiles of Lys-MIPs and Glu-MIPs, along with their non-imprinted controls, upon exposure to lysozyme and α-glucosidase. We evaluated the effect of a low enzyme concentrations, 0.000197% w/v, on the stability of the corresponding nanoparticles. This concentration is physiologically relevant, as it is comparable to lysozyme concentrations found in human serum and cerebrospinal fluid (CSF).^42^ The same concentration of α-glucosidase,^44^ however, was chosen arbitrarily, as it served primarily as a control to confirm the selective response of the imprinted nanoparticles to their specific enzyme template. The primary goal of this proof-of-concept study was to demonstrate a specific and significant response at a single, physiologically relevant enzyme concentration. A full investigation of the system's kinetics, including enzyme concentration-dependent release studies to determine site saturation, is a valuable direction for future work but is beyond the scope of the current manuscript.

As depicted in Fig. 5, Lys-MIPs exhibited a marked and progressive increase in size upon incubation with lysozyme in the dissolution medium. This swelling or aggregation behavior suggests a destabilization of the nanoparticle structure induced by the specific interaction of lysozyme with its imprinted sites. In contrast, Lys-MIPs incubated without lysozyme maintained a relatively stable size throughout the experimental period. Furthermore, non-imprinted nanoparticles (NINPs), whether exposed to lysozyme or incubated in buffer alone, showed no significant changes in size, indicating their stability and lack of specific interaction with the enzyme.

Similar trends were observed for Glu-MIPs. Exposure of Glu-MIPs to α-glucosidase resulted in a substantial increase in their hydrodynamic size over time, indicative of enzyme-triggered destabilization. Glu-MIPs in the absence of the enzyme and NINPs under both conditions, with or without α-glucosidase, remained stable in size. Interestingly, nanoparticle destabilization was not accompanied by a discernible change in polydispersity index (PDI), as in Fig. 5. Because PDI reflects population heterogeneity, its relative stability despite the marked size increase suggests that destabilization arises predominantly from a uniform swelling of individual particles rather than heterogeneous aggregation. While PDI alone cannot completely rule out subtle aggregation processes, the consistency of these findings with TEM and FTIR data (see below) supports the interpretation that recognition by the cognate enzyme induces a localized, imprint-guided structural disruption that enlarges particles while maintaining population uniformity.

**Fig. 6:**
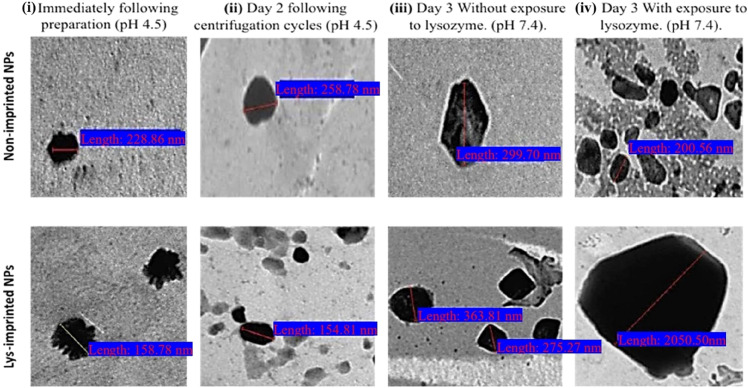
Transmission Electron Microscopy (TEM) images of lysozyme-imprinted nanoparticles (Lys-MIPs) and non-imprinted nanoparticles (NINPs) at different preparation and release stages.

To directly visualize the morphological consequences of this destabilization, Transmission Electron Microscopy (TEM) was employed (Fig. 6). NINPs consistently appeared as nearly spherical, electron-dense particles with relatively smooth surfaces and uniform size distributions. In contrast, Lys-MIPs displayed slightly rougher surfaces and discernible heterogeneity in their internal texture, consistent with the presence of imprinted cavities. When incubated under release conditions (pH 7.4, 37 °C), clear differences emerged. Lys-MIPs remained structurally intact in buffer without lysozyme. In contrast, exposure to lysozyme induced pronounced morphological alterations: particle outlines became less compact, partial shell loosening was evident, and regions of decreased electron density suggested internal destabilization. By comparison, NINPs exposed to lysozyme showed no appreciable morphological changes, confirming that destabilization is strictly imprint-guided and recognition-dependent. These TEM observations provide direct visual evidence that connects the macroscopic observation of swelling (DLS) with microscopic evidence of particle disruption, powerfully illustrating the concept of recognition-induced destabilization.

### 3.3. Functional consequence: specific, on-demand drug release

The functional consequence of enzyme-induced nanoparticle destabilization was assessed by examining the release profiles of ciprofloxacin at 37 °C. It is important to note that release experiments were conducted at pH 7.4 to mimic physiological conditions such as blood plasma and interstitial fluids, where therapeutic delivery is expected to occur. By contrast, nanoparticles were prepared at pH 4.5, a critical design choice to maintain enzyme inactivity during imprinting and to stabilize the CS-PH matrix during fabrication.^41–44^ Thus, preparation at acidic pH ensured nanoparticle integrity, whereas evaluation at neutral pH allowed us to assess enzyme-triggered destabilization and drug release under conditions that are biologically relevant.

**Fig. 7:**
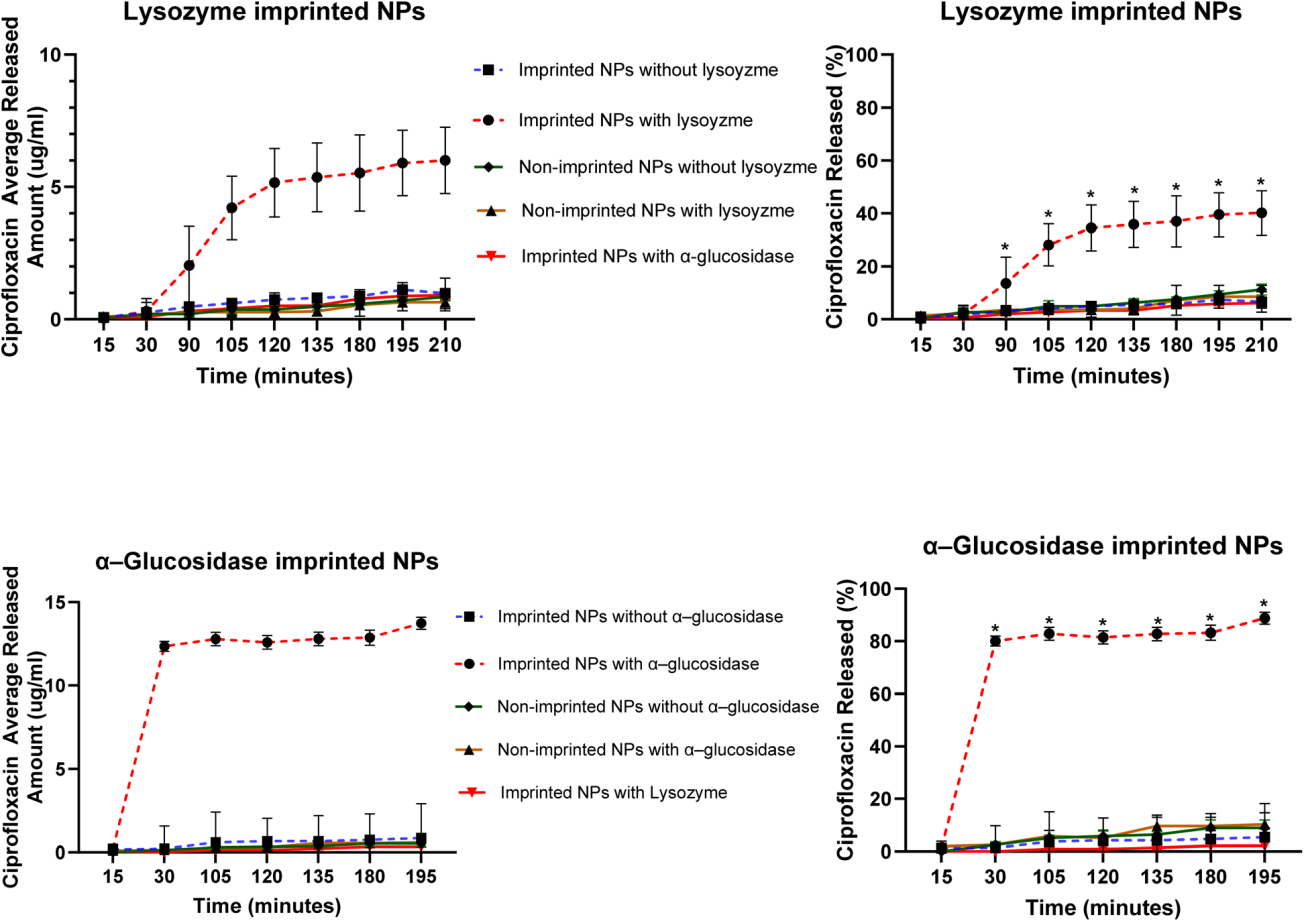
Release profiles of ciprofloxacin from imprinted NPs and non-imprinted NPs at 37 °C. Each point represents the average of three separate experiments (*n* = 3). Error bars represent the standard deviation of the measurements. A Student's *t*-test was used to determine statistical significance. Any release percentage data point annotated with * is significantly different from release percentages of all other nanoparticle formulations (*p* < 0.05).

Fig. 7 illustrates the cumulative amount and percentage of ciprofloxacin released from Lys-MIPs and Glu-MIPs under various conditions. For Lys-MIPs, exposure to lysozyme triggered a significantly accelerated and enhanced release of ciprofloxacin compared to all control groups. In the absence of lysozyme, or when Lys-MIPs were exposed to the non-template enzyme α-glucosidase, the drug release was minimal and comparable to that from NINPs exposed to lysozyme or NINPs in buffer alone. This demonstrates a specific enzyme-triggered release mechanism for Lys-MIPs.

A more pronounced effect was observed with Glu-MIPs. Upon incubation with α-glucosidase, Glu-MIPs released a substantial amount of ciprofloxacin, reaching approximately 90.46% of the encapsulated drug within 195 minutes. This is in stark contrast to the release from NINPs exposed to α-glucosidase, which released only about 10.27% of the drug under similar conditions. The specificity of the release was further confirmed by the low release from Glu-MIPs in the absence of α-glucosidase or in the presence of the non-template enzyme lysozyme. Non-imprinted nanoparticles in buffer alone also showed minimal drug release.

These results collectively indicate that the imprinted nanoparticles serve as efficient enzyme-responsive systems, releasing their payload predominantly in the presence of their specific target enzyme. The minimal drug release observed from the non-imprinted nanoparticles and the imprinted systems in the absence of their cognate enzyme (approximately 10% cumulative release over 195 minutes, Fig. 7) is a critical feature demonstrating the platform's high “off-state” stability. Ciprofloxacin (Fig. 1E) was deliberately selected as the model drug because its challenging physicochemical profile makes it a stringent test cargo for evaluating both stability and triggered release. Ciprofloxacin is a zwitterionic molecule with a well-documented U-shaped pH–solubility curve, exhibiting its lowest aqueous solubility in the neutral pH range.^50^ At the release medium pH of 7.4, it exists predominantly in its poorly soluble neutral form, which underlies its classification as a Biopharmaceutics Classification System (BCS) Class IV drug (low solubility/low permeability).^50^ This property ensures that passive leakage from the nanoparticles is minimized, thereby enabling us to probe whether enzyme-induced destabilization—not simple dissolution—drives drug release. Importantly, ciprofloxacin is also of high clinical relevance: it is a broad-spectrum fluoroquinolone widely used to treat severe infections, including those at sites where lysozyme and α-glucosidase are abundant (*e.g.*, respiratory tract, urinary tract, and inflamed tissues).^50^ Thus, using ciprofloxacin allows us to simultaneously validate the “off-state” robustness of our CS-PH nanoparticles and demonstrate their ability to achieve “on-demand” release of a poorly soluble yet therapeutically important antibiotic. The very shallow concentration gradient that develops from the drug's slow dissolution establishes a diffusion-locked state, which—according to Fick's law—severely hinders its release.^51^ In contrast, the rapid and extensive release from the enzyme-exposed MIPs (up to 90.46%) shows that recognition-induced matrix destabilization is essential to unlock this diffusion barrier, thereby enabling efficient, biologically triggered delivery.

The functional consequence of enzyme-induced destabilization was evident in the ciprofloxacin release studies. Imprinted nanoparticles exhibited markedly accelerated and enhanced drug release only in the presence of their specific enzyme, whereas non-imprinted controls and off-target enzyme exposures showed minimal release. This selective, enzyme-triggered response highlights the “on-demand” functionality imparted by molecular imprinting. The release was highly specific; the drug was retained within the MIPs without the enzymatic trigger or in the presence of a non-template enzyme. This minimal leakage is crucial for preventing premature release and off-target effects. The observed destabilization is directly linked to the enhanced drug release, as the swelling of the nanoparticle structure likely increases matrix porosity, facilitating drug diffusion. Thus, molecular imprinting transforms CS-PH into a specific, enzyme-responsive platform, where the imprinting process imparts a “smart” functionality that allows the nanoparticles to sense and respond to a biological signal. This system is promising for applications requiring drug release at sites with specific enzyme activity.

### 3.4. Elucidating the molecular mechanism of destabilization

**Fig. 8:**
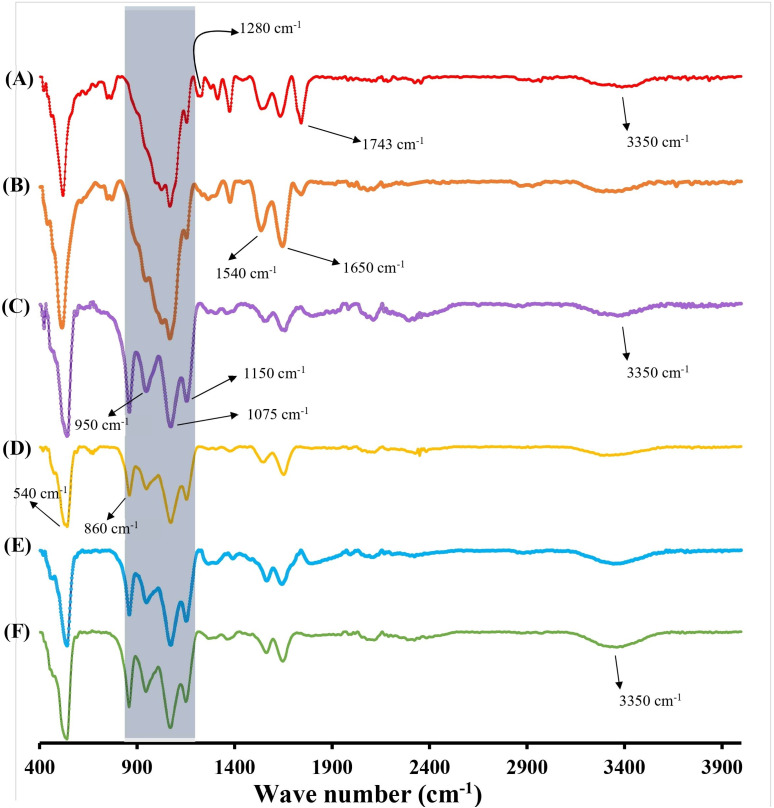
IR spectra of various NPs. (A) Non-imprinted NPs lyophilized immediately after preparation at pH 4.5 and 25 °C. (B) Lysozyme-imprinted NPs lyophilized immediately after preparation at pH 4.5 and 25 °C. (C) Lysozyme-imprinted NPs lyophilized after 2 hours of incubation in the dissolution medium at pH 7.4 and 37 °C. (D) Lysozyme-imprinted NPs lyophilized after 2 hours of incubation with lysozyme in the dissolution medium at pH 7.4 and 37 °C. (E) Non-imprinted NPs lyophilized after 2 hours of incubation in the dissolution medium at pH 7.4 and 37 °C. (F) Non-imprinted NPs lyophilized after 2 hours of incubation with lysozyme in the dissolution medium at pH 7.4 and 37 °C. Note the subtle but distinct decrease in the relative intensity of the absorption band region from 1150 cm^−1^ to 860 cm^−1^ in spectrum (D) compared to controls (C, E and F) (highlighted with shading). This region is characteristic of the C–O–C stretching of the β-1,4-glycosidic linkages, and its reduction provides direct chemical evidence of imprint-guided, localized degradation of the chitosan backbone.

To probe the underlying chemical changes driving destabilization, FTIR spectra were recorded for the nanoparticle formulations (Fig. 8). The spectra of NINPs (Fig. 8A) and Lys-MIPs (Fig. 8B) confirmed the successful formulation of the crosslinked chitosan-phthalate (CS-PH) matrix. Both spectra display the characteristic broad O–H/N–H stretching band (∼3350 cm^−1^), amide I and II bands (1650 cm^−1^ and 1540 cm^−1^, respectively), and the complex C–O–C stretching vibrations of the polysaccharide backbone (1150–860 cm^−1^). The sequential ionotropic/covalent crosslinking with TPP and EDC is indicated by bands characteristic of N–O–P linkages (see Fig. 8A) at 950 and 1280 cm^−1^.^29,30^ A distinct shoulder at 1743 cm^−1^, attributed to the C=O stretch of protonated phthalate carboxyl groups, is present in the nanoparticles at their acidic preparation pH of 4.5. As expected, this peak disappears in all samples incubated at pH 7.4 (Fig. 8C–F), confirming the deprotonation of these groups and the structural integrity of the matrix upon pH change.

The critical insight from the FTIR analysis arises from comparing nanoparticles incubated with and without lysozyme. The spectrum of Lys-MIPs exposed to lysozyme (Fig. 8D) reveals a subtle but distinct decrease in the relative intensity of the absorption band region from 1150 cm^−1^ to 860 cm^−1^ (*e.g.*, compared to amide I and II bands at ∼1650 cm^−1^ and 1540 cm^−1^, respectively). This region is characteristic of the C–O–C stretching of the β-1,4-glycosidic linkages that form the chitosan backbone. This reduction suggests a partial cleavage of these bonds, providing direct chemical evidence of subtle enzymatic degradation. Crucially, this spectral change is highly specific. It is not observed in the control spectrum of Lys-MIPs incubated without the enzyme (Fig. 8C), nor is it apparent in the spectrum of NINPs exposed to lysozyme (Fig. 8F). The absence of significant degradation in NINPs indicates that random, non-specific enzyme adsorption is insufficient to induce cleavage. Therefore, the data strongly supports the hypothesis that the molecular imprints act as recognition sites, guiding the enzyme to facilitate a localized degradation of the nanoparticle matrix.

This specific, imprint-mediated chemical change provides the basis for the destabilization mechanism. A central hypothesis of this study was that molecularly imprinting chitosan nanoparticles with digestive enzymes would create specific recognition sites. The fabrication process utilized inactive enzymes at an acidic pH of 4.5, which, after removal, left behind specific binding sites. At a physiological pH of 7.4, the now-active enzymes bind to these sites, leading to nanoparticle degradation and triggered drug release. Dynamic Light Scattering (DLS) data strongly supports this, showing a significant, time-dependent increase in the size of imprinted nanoparticles (Lys-MIPs and Glu-MIPs) only when exposed to their corresponding enzymes, indicating structural destabilization. This response was highly specific, as non-imprinted nanoparticles (NINPs) and imprinted nanoparticles in the absence of their target enzyme showed no such changes. This selective destabilization suggests a “lock-and-key” mechanism, confirming the creation of functional molecular imprints.

We propose that this destabilization is driven by a proximity-induced effect, a concept well-established in fields like nanocatalysis.^52–54^ The specific binding of the enzyme within the sterically confined imprinted cavity forces a prolonged, high-proximity interaction between the enzyme's active site and the chitosan backbone. This “proximity effect” could induce significant conformational strain on the glycosidic bonds, lowering the activation energy for a localized hydrolytic cleavage. This targeted cleavage would be kinetically inaccessible to enzymes interacting *via* random, transient adsorption on the non-imprinted surface, thus explaining the high specificity of the observed destabilization. This process is not random swelling but a targeted, programmed response based on specific molecular recognition, highlighting the “smart” nature of the system.

Although direct visualization of amorphous, nanoscale cavities is inherently challenging, multiple lines of evidence confirm the successful formation of functional imprints. Functionally, only imprinted nanoparticles exhibited enzyme-triggered destabilization (Fig. 5) and drug release (Fig. 7), demonstrating a clear “lock-and-key” response. Physicochemical data reinforce this conclusion: imprinted nanoparticles displayed a twofold increase in drug loading (Table 2), markedly lower PDI values indicative of template-guided assembly (Table 1), and AFM imaging revealed more textured surfaces consistent with imprint-induced features (Fig. 3), while DSC thermograms showed reproducible reductions in melting temperature of imprinted systems (Fig. 4), reflecting subtle matrix destabilization accompanying the introduced vacant surface imprints.

TEM analysis (Fig. 6) further corroborated these findings, showing that imprinted nanoparticles retained spherical morphology but exhibited surface roughness and, critically, underwent structural loosening and reduced electron density upon lysozyme exposure, in agreement with PDI data that indicated uniform destabilization rather than heterogeneous aggregation (Fig. 5). FTIR spectra revealed selective reductions in the C–O–C stretching region upon enzyme exposure, confirming localized backbone disruption (Fig. 8). Collectively, these functional, morphological, and spectroscopic results—including the direct visualization from TEM—establish that molecular imprinting confers selective recognition, enzyme-triggered destabilization, and enhanced drug loading, underscoring its promise for advanced enzyme-responsive drug delivery systems.

### 3.5. Mechanistic discussion

The mechanism underlying recognition-induced destabilization differs fundamentally from conventional enzyme-responsive drug delivery systems. Traditionally, enzyme-triggered nanoparticle degradation relies on catalytic cleavage of polymer backbones or crosslinks, gradually weakening the material until disassembly occurs.^8,9^ While effective, this approach has inherent limitations: enzymatic hydrolysis is relatively slow, often incomplete, and prone to cross-reactivity since many enzymes share overlapping substrate affinities.^8,9^ Consequently, degradation-based systems may exhibit partial instability under physiological conditions, leading to unwanted basal release and reduced on/off control.

By contrast, the present system harnesses molecular recognition alone as the destabilization trigger. In our design, imprinting creates cavities with precise chemical and steric complementarity to the target enzyme. Upon binding, the enzyme exerts localized conformational strain and disrupts the tightly crosslinked CS-PH network. Importantly, this destabilization does not require catalytic turnover, but instead arises from proximity-driven microstructural stress within the imprinted cavity. This “lock-and-key destabilization” enables a sharp and amplified transition from a highly stable “off-state” to a rapid release “on-state.” Several advantages emerge from this recognition-driven mechanism:

(1) Specificity: imprint-guided binding restricts nanoparticle destabilization to the cognate enzyme, whereas cleavage-dependent systems may respond to multiple enzymes with shared substrate profiles. Our results confirm this point—non-template enzymes failed to trigger release (Fig. 5 and 7), highlighting the molecular precision of the system.

(2) Rapid response: the abrupt release (>90% within 195 minutes) highlights the efficiency of this mechanism, contrasting with the gradual and diffusion-limited release typical of degradation-based systems. Because destabilization is recognition-triggered rather than hydrolysis-limited, the kinetics are faster and more controllable.

(3) Stringent baseline stability: dual crosslinking confers extraordinary inherent stability to the CS-PH nanoparticles, as reflected in the negligible basal release (<11%). This makes the enzyme-triggered “on-state” response more distinct and reliable than many conventional systems, where partial swelling or nonspecific hydrolysis contributes to background leakage.

(4) Broader applicability: recognition-induced destabilization is not restricted to enzymes capable of cleaving chitosan. Indeed, both lysozyme and α-glucosidase—enzymes incapable of catalyzing chitosan degradation—successfully triggered disassembly. This expands the scope of enzyme-responsive drug delivery beyond degradative enzymes to any protein biomarker that can be imprinted, thereby significantly broadening potential biomedical applications.

When compared with other classes of stimuli-responsive systems—such as pH-, redox-, or temperature-sensitive carriers—our recognition-induced destabilization offers a much higher level of “address resolution.” Traditional systems respond to broad, non-specific environmental changes that can occur in both healthy and diseased tissues, whereas molecular imprinting enables programmed responses to discrete biological signals. In this respect, recognition-induced destabilization provides not only a new mechanism of nanoparticle breakdown but also a powerful strategy for designing highly specific smart materials.

Our findings further highlight the stringent baseline stability of CS-PH nanoparticles under physiological conditions (pH 7.4, 37 °C). In the absence of the cognate enzyme, both imprinted and non-imprinted nanoparticles maintained their integrity and released less than ∼11% of ciprofloxacin over 195 minutes, underscoring their robustness against nonspecific degradation or premature leakage. This stability is critical for potential biomedical applications, where nanoparticles are expected to circulate or reside in enzyme-rich physiological environments without significant drug loss until encountering their intended trigger. Importantly, lysozyme- and α-glucosidase-imprinted nanoparticles achieved selective and accelerated drug release only in the presence of their respective enzymes, mimicking conditions likely encountered at infection or inflammation sites where these enzymes are overexpressed. Such stability–responsiveness duality makes the system highly attractive for applications that demand precise spatiotemporal control of drug delivery. For instance, lysozyme-responsive systems may be useful for targeting infections in the respiratory tract or urinary tract, while α-glucosidase-responsive systems could be adapted for gastrointestinal delivery or for therapeutic modulation in metabolic disorders where this enzyme plays a key role. Beyond drug delivery, the platform could be extended to diagnostic or biosensing applications, where recognition-induced destabilization of nanoparticles can serve as an amplified readout for detecting disease-associated enzymes.

## 4. Conclusion

In conclusion, this work establishes and validates a new paradigm for the design of smart materials, termed recognition-induced destabilization. Unlike conventional enzyme-responsive systems, which rely on catalytic cleavage of polymer backbones and often suffer from slow kinetics, partial specificity, and baseline instability, our approach transforms molecular recognition itself into a direct trigger for nanoparticle disassembly. By imprinting enzymes into a highly stable CS-PH matrix, we demonstrated that specific “lock-and-key” binding can impose proximity-driven microstructural strain, leading to selective destabilization and rapid drug release.

This mechanism offers several distinct advantages: (i) exceptional specificity, as only cognate enzymes induce destabilization, while non-template enzymes fail to trigger release; (ii) rapid and amplified responsiveness, with abrupt transitions from diffusion-locked “off-states” to extensive drug release “on-states” (>90% within 195 minutes); (iii) stringent stability baseline, since dual-crosslinked CS-PH nanoparticles remain highly resistant to nonspecific degradation and premature leakage (<11% release without enzyme); and (iv) broader adaptability, as recognition-induced destabilization does not depend on enzymatic cleavage, thereby expanding applicability to a wide range of non-degradative biomarker proteins.

When compared to other stimuli-responsive strategies (pH, redox, temperature), recognition-induced destabilization achieves a much higher level of “address resolution,” responding only to discrete biomolecular cues rather than general environmental changes. This high-fidelity recognition-to-response pathway not only validates its promise for targeted drug delivery but also lays the foundation for developing next-generation smart materials in biosensing, diagnostics, and controlled catalysis.

Translating this platform into *in vivo* applications offers exciting opportunities but also presents important challenges that will guide the next phase of research. Key considerations include mitigating protein corona formation in biological fluids, which may otherwise shield the imprinted recognition sites; minimizing potential immunogenicity of the nano-MIPs; and optimizing stability and pharmacokinetics in complex biological environments. Addressing these aspects not only represents essential steps toward clinical translation but also provides opportunities to further refine and expand the versatility of this recognition-induced destabilization platform.

## Author contributions

Mutasem O. Taha: conceptualization, methodology, supervision, writing – original draft, writing – review & editing, funding acquisition. Isra Dmour: methodology, investigation, writing – review & editing. Ramzi Mukred Saeed: investigation, visualization, writing – review & editing. Taqwa Alfararjeh: investigation, data curation. Inshad Jum'h: investigation (AFM), formal analysis. Lina A Dahabiyeh: resources. All authors have read and agreed to the published version of the manuscript.

## Conflicts of interest

There are no conflicts of interest to declare.

## Supplementary Material

RA-015-D5RA05081B-s001

## Data Availability

All data supporting the findings of this study are available within the paper and supplementary information (SI). Supplementary information is available. See DOI: https://doi.org/10.1039/d5ra05081b.

## References

[cit1] Cook A., Decuzzi P. (2021). Harnessing Endogenous Stimuli for Responsive Materials in Theranostics. ACS Nano.

[cit2] Rahim M., Jan N., Khan S., Shah H., Madni A., Khan A., Jabar A., Khan S., Elhissi A., Hussain Z., Aziz H., Sohail M., Khan M., Thu H. (2021). Recent Advancements in Stimuli Responsive Drug Delivery Platforms for Active and Passive Cancer Targeting. Cancers.

[cit3] Mura S., Nicolas J., Couvreur P. (2013). Stimuli-responsive nanocarriers for drug delivery. Nat. Mater..

[cit4] Miao Y., Feng Y., Bai J., Liu Z., Zhao X. (2021). Optimized mesoporous silica nanoparticle-based drug delivery system with removable manganese oxide gatekeeper for controlled delivery of doxorubicin. J. Colloid Interface Sci..

[cit5] Wang X., Yao H., Li X., Wang X., Huang Y., Liu Z. (2016). pH/temperature-sensitive hydrogel-based molecularly imprinted polymers (hydroMIPs) for drug delivery by frontal polymerization. RSC Adv..

[cit6] Wang X., Yang F., Zhang L., Huang Y., Liu Z. (2018). A polyhedral oligomeric silsesquioxane/molecular sieve codoped molecularly imprinted polymer for gastroretentive drug-controlled release in vivo. Biomater. Sci..

[cit7] Foster D., Cakley A., Larsen J. (2024). Optimizing enzyme-responsive polymersomes for protein-based therapies. Nanomedicine.

[cit8] Sobczak M. (2022). Enzyme-responsive hydrogels as potential drug delivery systems—state of knowledge and future prospects. Int. J. Mol. Sci..

[cit9] Li M., Zhao G., Su W.-K., Shuai Q. (2020). Enzyme-responsive nanoparticles for anti-tumor drug delivery. Front. Chem..

[cit10] Choi J., Yong K., Choi J., Cowie A. (2019). Progress in Molecularly Imprinted Polymers for Biomedical Applications. Comb. Chem. High Throughput Screen..

[cit11] BelBruno J. (2019). Molecularly Imprinted Polymers. Chem. Rev..

[cit12] Liu R., Poma A. (2021). Advances in Molecularly Imprinted Polymers as Drug Delivery Systems. Molecules.

[cit13] Liu X., Zhou T., Du Z., Wei Z., Zhang J. (2011). Recognition ability of temperature responsive molecularly imprinted polymer hydrogels. Soft Matter.

[cit14] Qin L., He X., Yuan X., Li W., Zhang Y. (2011). Molecularly imprinted beads with double thermosensitive gates for selective recognition of proteins. Anal. Bioanal. Chem..

[cit15] Suksuwan A., Lomlim L., Rungrotmongkol T., Nakpheng T., Dickert F., Suedee R. (2015). The composite nanomaterials containing (R)-thalidomide-molecularly imprinted polymers as a recognition system for enantioselective-controlled release and targeted drug delivery. J. Appl. Polym. Sci..

[cit16] Li C., Ma Y., Niu H., Zhang H. (2015). Hydrophilic Hollow Molecularly Imprinted Polymer Microparticles with Photo- and Thermoresponsive Template Binding and Release Properties in Aqueous Media. ACS Appl. Mater. Interfaces.

[cit17] Gong C., Lam M., Yu H. (2006). The Fabrication of a Photoresponsive Molecularly Imprinted Polymer for the Photoregulated Uptake and Release of Caffeine. Adv. Funct. Mater..

[cit18] Liu L., Chen M., Yang H., Huang Z., Tang Q., Chow C., Gong C., Zu M., Xiao B. (2020). An NIR-light-responsive surface molecularly imprinted polymer for photoregulated drug release in aqueous solution
through porcine tissue. Mater. Sci. Eng., C.

[cit19] Zhang K., Guan X., Qiu Y., Wang D., Zhang X., Zhang H. (2016). A pH/glutathione double responsive drug delivery system using molecular imprint technique for drug loading. Appl. Surf. Sci..

[cit20] Liu S., Bi Q., Long Y., Li Z., Bhattacharyya S., Li C. (2017). Inducible epitope imprinting: 'generating' the required binding site in membrane receptors for targeted drug delivery. Nanoscale.

[cit21] Cecchini A., Raffa V., Canfarotta F., Signore G., Piletsky S., MacDonald M., Cuschieri A. (2017). In Vivo Recognition of Human Vascular Endothelial Growth Factor by Molecularly Imprinted Polymers. Nano Lett..

[cit22] Canfarotta F., Lezina L., Guerreiro A., Czulak J., Petukhov A., Daks A., Smolinska-Kempisty K., Poma A., Piletsky S., Barlev N. (2018). Specific Drug Delivery to Cancer Cells with Double-Imprinted Nanoparticles against Epidermal Growth Factor Receptor. Nano Lett..

[cit23] Zhang Y., Deng C., Liu S., Wu J., Chen Z., Li C., Lu W. (2015). Active targeting of tumors through conformational epitope imprinting. Angew Chem. Int. Ed. Engl..

[cit24] Dong Y., Li W., Gu Z., Xing R., Ma Y., Zhang Q., Liu Z. (2019). Inhibition of HER2-Positive Breast Cancer Growth by Blocking the HER2 Signaling Pathway with HER2-Glycan-Imprinted Nanoparticles. Angew Chem. Int. Ed. Engl..

[cit25] Medina Rangel P., Moroni E., Merlier F., Gheber L. A., Vago R., Tse Sum Bui B., Haupt K. (2020). Chemical Antibody Mimics Inhibit Cadherin-Mediated Cell-Cell Adhesion: A Promising Strategy for Cancer Therapy. Angew Chem. Int. Ed. Engl..

[cit26] Bărăian A.-I., Iacob B.-C., Bodoki A. E., Bodoki E. (2022). In vivo applications of molecularly imprinted polymers for drug delivery: a pharmaceutical perspective. Int. J. Mol. Sci..

[cit27] Zaidi S. A. (2016). Latest trends in molecular imprinted polymer based drug delivery systems. RSC Adv..

[cit28] Xu S., Wang L., Liu Z. (2021). Molecularly Imprinted Polymer Nanoparticles: An Emerging Versatile Platform for Cancer Therapy. Angew Chem. Int. Ed. Engl..

[cit29] Dmour I., Taha M. (2017). Novel nanoparticles based on chitosan-dicarboxylate conjugates via tandem ionotropic/covalent crosslinking with tripolyphosphate and subsequent evaluation as drug delivery vehicles. Int. J. Pharm..

[cit30] Saeed R., Dmour I., Taha M. (2020). Stable chitosan-based nanoparticles using polyphosphoric acid or hexametaphosphate for tandem ionotropic/covalent crosslinking and subsequent investigation as novel vehicles for drug delivery. Front. Bioeng. Biotechnol..

[cit31] Haider A., Khan S., Iqbal D. N., Shrahili M., Haider S., Mohammad K., Mohammad A., Rizwan M., Kanwal Q., Mustafa G. (2024). Advances in chitosan-based drug delivery systems: A comprehensive review for therapeutic applications. Eur. Polym. J..

[cit32] Alves N., Mano J. (2008). Chitosan derivatives obtained by chemical modifications for biomedical and environmental applications. Int. J. Biol. Macromol..

[cit33] Bracharz F., Helmdach D., Aschenbrenner I., Funck N., Wibberg D., Winkler A., Bohnen F., Kalinowski J., Mehlmer N., Brück T. (2018). Harvest of the oleaginous microalgae Scenedesmus obtusiusculus by flocculation from culture based on natural water sources. Front. Bioeng. Biotechnol..

[cit34] Deng P., Jin W., Liu Z., Gao M., Zhou J. (2021). Novel multifunctional adenine-modified chitosan dressings for promoting wound healing. Carbohydr. Polym..

[cit35] Jiang Y., Wu J. (2019). Recent development in chitosan nanocomposites for surface-based biosensor applications. Electrophoresis.

[cit36] Park M., Li M., Yeo I., Jung J., Yoon B., Joung Y. (2020). Balanced adhesion and cohesion of chitosan matrices by conjugation and oxidation of catechol for high performance surgical adhesives. Carbohydr. Polym..

[cit37] Rasul R., Tamilarasi Muniandy M., Zakaria Z., Shah K., Chee C., Dabbagh A., Rahman N., Wong T. (2020). A review on chitosan and its development as pulmonary particulate anti-infective and anti-cancer drug carriers. Carbohydr. Polym..

[cit38] Thakur C., Thotakura N., Kumar R., Kumar P., Singh B., Chitkara D., Raza K. (2016). Chitosan-modified PLGA polymeric nanocarriers with better delivery potential for tamoxifen. Int. J. Biol. Macromol..

[cit39] Silva M., Calado R., Marto J., Bettencourt A., Almeida A., Gonçalves L. (2017). Chitosan nanoparticles as a mucoadhesive drug delivery system for ocular administration. Mar. Drugs.

[cit40] Dmour I., Taha M. O. (2023). Tableting-induced mechanochemical matrix crosslinking: Towards non-disintegrating chitosan-based sustained delivery tablets. J. Drug Delivery Sci. Technol..

[cit41] Callewaert L., Michiels C. (2010). Lysozymes in the animal kingdom. J. Biosci..

[cit42] Ragland S., Criss A. (2017). From bacterial killing to immune modulation: Recent insights into the functions of lysozyme. PLoS Pathog..

[cit43] Islam N., Dmour I., Taha M. O. (2019). Degradability of chitosan micro/nanoparticles for pulmonary drug delivery. Heliyon.

[cit44] Okuyama M., Saburi W., Mori H., Kimura A. (2016). α-Glucosidases and α-1,4-glucan lyases: structures, functions, and physiological actions. Cell. Mol. Life Sci..

[cit45] Aiedeh K., Taha M. O. (1999). Synthesis of chitosan succinate and chitosan phthalate and their evaluation as suggested matrices in orally administered, colon-specific drug delivery systems. Arch. Pharm..

[cit46] Calvo P., Remunan-Lopez C., Vila-Jato J., Alonso M. (1997). Chitosan and chitosan/ethylene oxide-propylene oxide block copolymer nanoparticles as novel carriers for proteins and vaccines. Pharm. Res..

[cit47] Werner M., Glück M., Bräuer B., Bismarck A., Lieberzeit P. (2022). Investigations on sub-structures within cavities of surface imprinted polymers using AFM and PF-QNM. Soft Matter.

[cit48] Fresco-Cala B., Batista A. D., Cárdenas S. (2020). Molecularly imprinted polymer micro- and nano-particles: a review. Molecules.

[cit49] Rosengren A. M., Karlsson B. C., Nicholls I. A. (2013). Consequences of morphology on molecularly imprinted polymer–ligand recognition. Int. J. Mol. Sci..

[cit50] Olivera M. E., Manzo R. H., Junginger H. E. (2011). *et al.*, Biowaiver monographs for immediate release solid oral dosage forms: ciprofloxacin hydrochloride. J. Pharm. Sci..

[cit51] Heredia N. S., Vizuete K., Flores-Calero M., Pazmiño V. K., Pilaquinga F. (2022). *et al.*, Comparative statistical analysis of the release kinetics models for nanoprecipitated drug delivery systems based on poly(lactic-co-glycolic acid). PLoS One.

[cit52] Bruice T. C. , Proximity effects and enzyme catalysis, in The Enzymes, ed. P. D. Boyer, Academic Press, 1970, vol. 2, pp. 217–279

[cit53] Kinghorn M. J., Valdivia-Berroeta G. A., Chantry D. R., Smith M. S., Ence C. C., Draper S. R. E., Duval J. S., Masino B. M., Cahoon S. B., Flansburg R. R., Conder C. J., Price J. L., Michaelis D. J. (2017). Proximity-induced reactivity and product selectivity with a rationally designed bifunctional peptide catalyst. ACS Catal..

[cit54] Lim K. R. G., Kaiser S. K., Wu H., Garg S., O'Connor C. R., Reece C., Aizenberg M., Aizenberg J. (2024). Deconvoluting the individual effects of nanoparticle proximity and size in thermocatalysis. ACS Nano.

