# Supplementary material for: Recognition-induced destabilization: controlled release from molecularly imprinted chitosan nanoparticles via specific, non-catalytic enzyme recognition
Source: RSC Adv. 2025 Oct 31;15(50):42315–30. doi: 10.1039/d5ra05081b (PMC12577715; doi:10.1039/d5ra05081b)
Supplement: RA-015-D5RA05081B-s001 [file RA-015-D5RA05081B-s001.pdf]

Supplementary Information (SI) for RSC Advances.
This journal is © The Royal Society of Chemistry 2025

Supplementary Materials

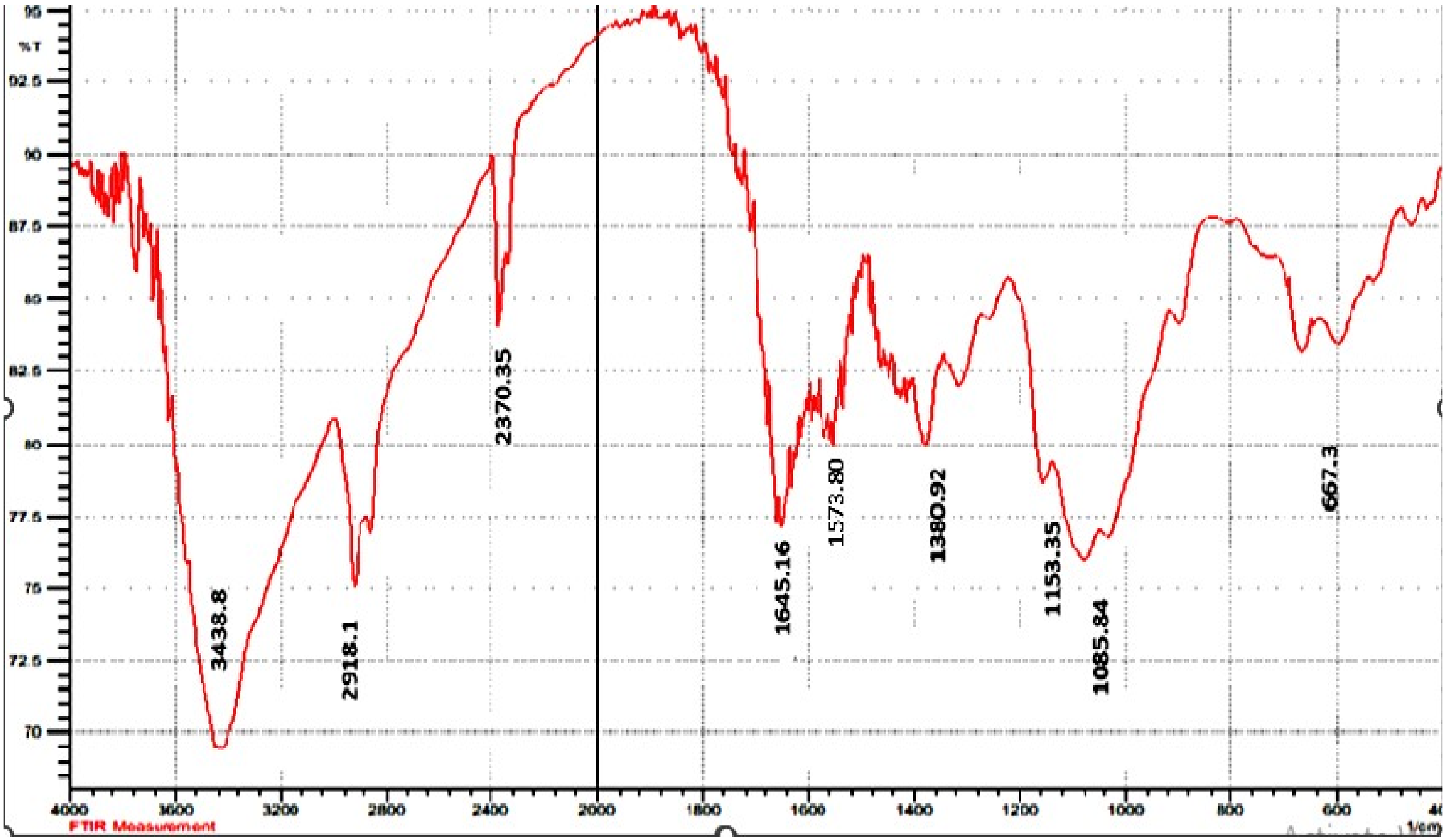

Figure S1: Infrared of Chitosan (C).

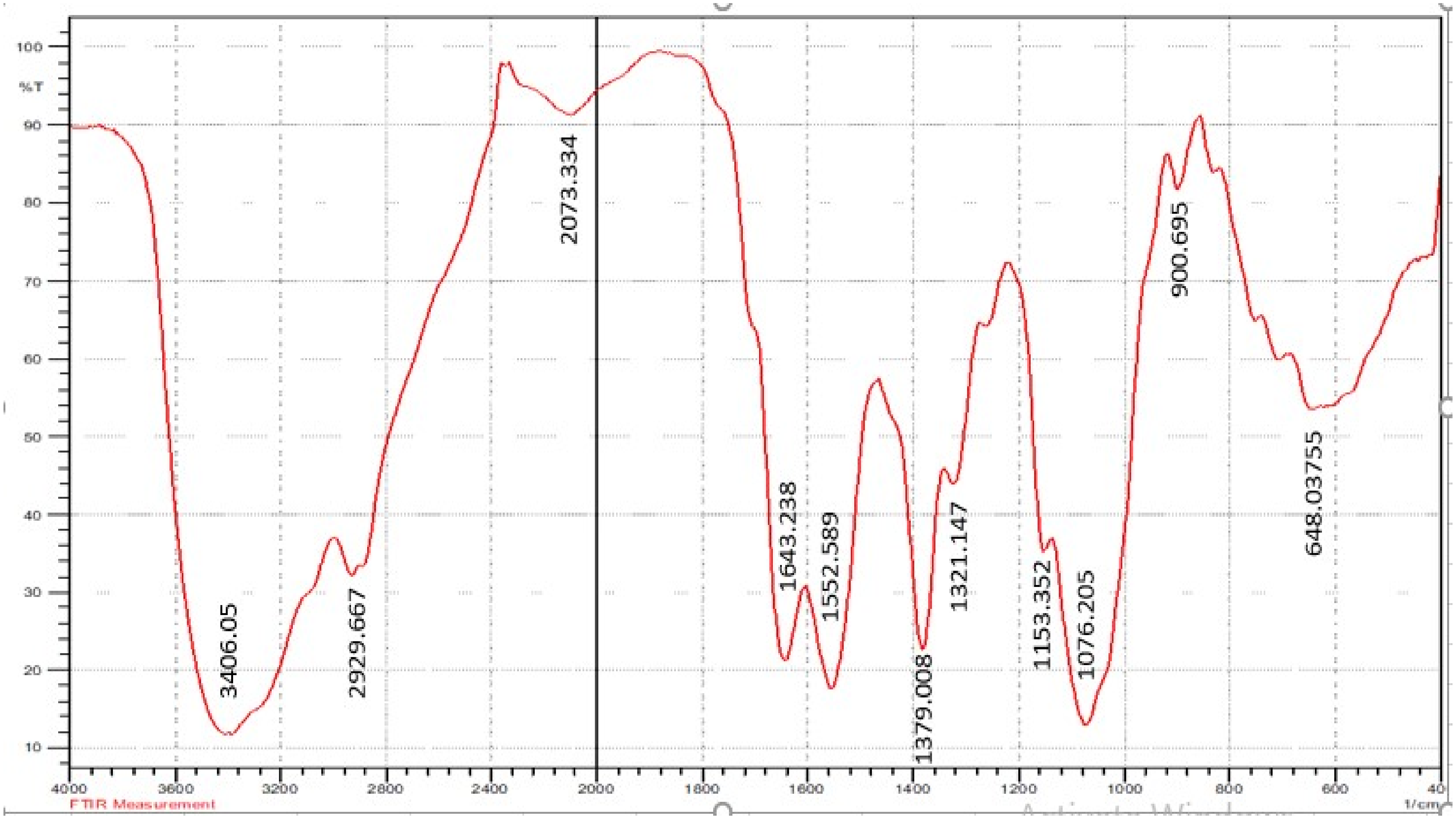

Figure S2: Infrared of Chitosan Phthalate (CP).

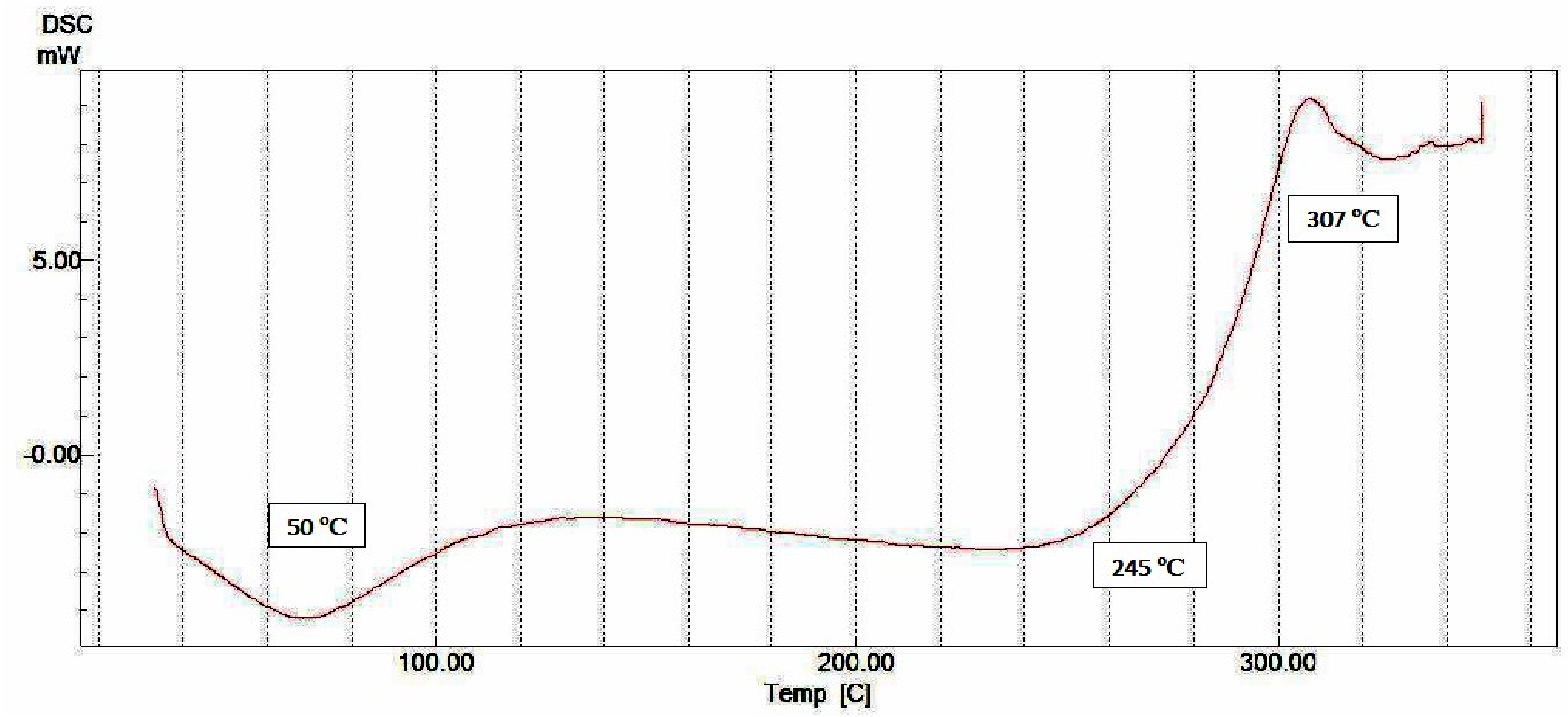

Figure S3: DSC of Chitosan (C).

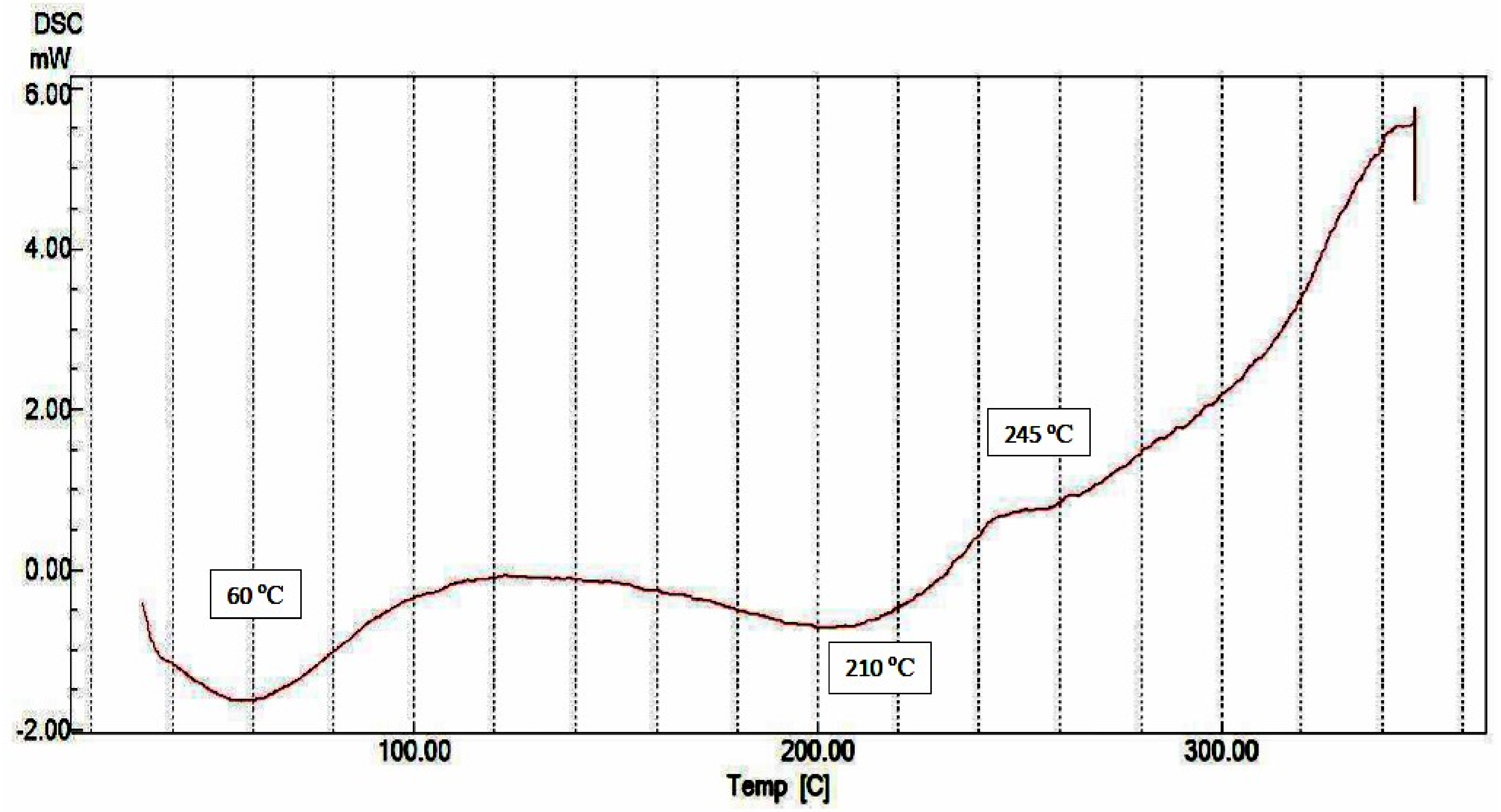

Figure S4: DSC of Chitosan Phthalate (CP)

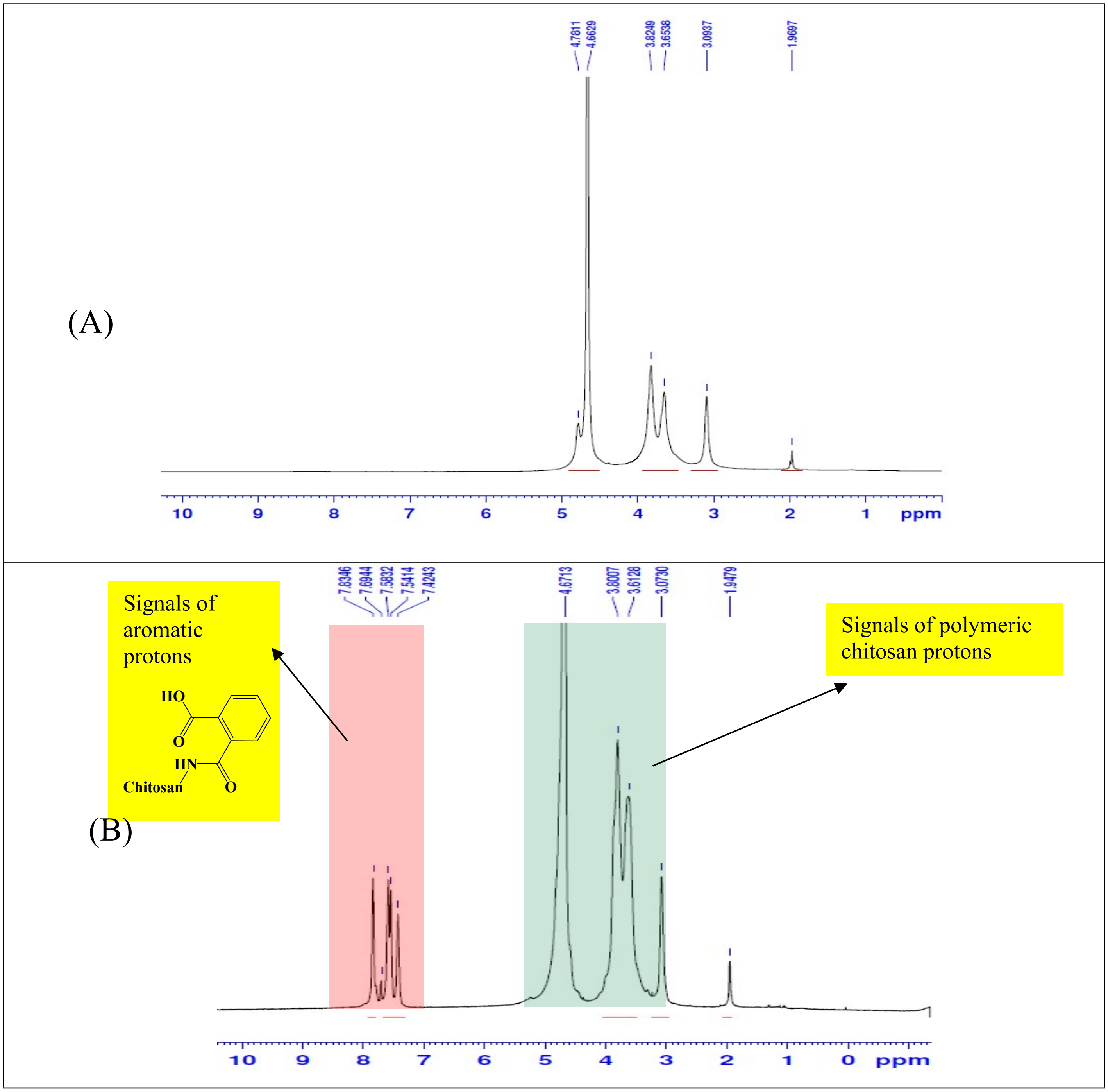

Figure S5 ^{1}H NMR spectrums of **(A)** chitosan, **(B)** chitosan phthalate.
